# Numerical Analysis of the Combustion of Gases Generated during Biomass Carbonization

**DOI:** 10.3390/e22020181

**Published:** 2020-02-05

**Authors:** Robert Zarzycki, Rafał Kobyłecki, Zbigniew Bis

**Affiliations:** Department of Energy Engineering, Faculty of Environmental Engineering and Biotechnology, Czestochowa University of Technology, 42-201 Częstochowa, Poland; zarzycki@is.pcz.pl (R.Z.); zbis@is.pcz.pl (Z.B.)

**Keywords:** thermolysis, pyrolysis, biomass, biochar, biomass gasification, polygeneration

## Abstract

The paper deals with the analysis of the combustion of volatiles evolved during thermolysis (thermal treatment) of biomass feedstock. The process is tailored to produce charcoal (biochar), heat and electricity and the whole system consists of a carbonizer, afterburning chamber and steam recovery boiler. In order to maintain safe operation of the carbonizer the process temperature has to be maintained at an acceptable level and thus the majority of gases evolved during biomass processing have to be combusted outside in the afterburning chamber. In this paper the combustion of those gases in a specially-designed combustion chamber was investigated numerically. The calculation results indicated that the production of the biochar has to be carried out with tight integration and management of the heat produced from the combustion of the volatiles and the emission of CO and methane may be maintained at a low level by optimization of the combustion process. The most promising effects were achieved in cases C4 and C5 where the gas was fed tangentially into the afterburning chamber. The calculation results were then used for the design and manufacture of a pilot reactor—from which the parameters and operational data will be presented and discussed in a separate paper.

## 1. Introduction

In recent years, climate change has been observed in the form of violent atmospheric phenomena (storms, tornadoes). There is also a continuous increase in average world temperatures, which results in droughts and, consequently, may cause problems with food production in many countries. The increase in average air temperature causes the melting of glaciers, which leads to flooding of the parts of the land with the lowest altitudes. These phenomena are partly due to the intensive development of the industry, transport and fossil fuel energy sectors. Furthermore, climate change is also affected by natural phenomena such as volcanic eruptions and solar activity. In order to slow down or stop climate change, it is necessary to significantly reduce the use of fossil fuels and replace them with renewable energy [1]. Renewable energy can be analyzed within several categories, but the most important is its predictability related to the security of energy supply. Solar and wind-based power generation is highly dependent on weather conditions, which does not ensure the continuity of electricity supply. Renewable power generation based on the use of potential energy of water, tides, geothermal energy and biomass combustion allows for ensuring constant energy supply to meet the current needs. Therefore, it is necessary to develop those sectors of renewable energy generation which allow for continuity of the electricity supply. One of the ways to accomplish this is the use of technology for converting biomass into charcoal, combined with the production of electricity [2]. Such an approach seems to be particularly promising for Poland where the agricultural sector is quite strong and may supply significant amount of feedstock for sustainable and environmentally friendly electricity generation [3].

As is well known, biomass is defined as the biodegradable fraction of products, waste or residues of biological origin from agriculture and forestry in the form of wood chips, briquettes or pellets. Biomass can be used for energy purposes by burning it in dedicated power equipment (boilers) to produce heat and then generate electricity. Biomass can also be subjected to thermal processing and carbonization in dedicated reactors—the main goal of such treatment is the removal of moisture from biomass and transformation of the feedstock into an environmentally stable product that may then be used as a sorbent, water retention agent, soil component, or feedstock for some more advanced processes (e.g., for hydrogen production) [3]. It is possible to produce charcoal, and, by burning biomass devolatilization products, to produce heat and electricity [4,5,6]. The charcoal production process is carried out in dedicated reactors, most often with continuous operation. Reactors of this type are made of a pipe with diameter from 100 to 800 mm, with an auger ensuring the transportation of biomass during its thermal treatment and transformation into charcoal. There are holes along the pipe allowing gases to flow outside, where their partial combustion is carried out in a controlled way, and the released heat maintains the process of heating, drying and devolatilization of the newly supplied biomass and its continuous carbonization [7]. Depending on the type and water content of the carbonized biomass, the amount of energy contained in the released gases during the thermal processing of the feedstock may significantly exceed the amount of heat necessary to maintain the process [8]. In such cases, in order to ensure safe operation of the reactor (i.e., to maintain the temperature inside the reactor at a safe level), it is necessary to burn some of the excess gases in a dedicated combustion chamber outside the carbonization reactor. This paper presents numerical calculations of the combustion chamber designed for the burning of the gases produced during biomass thermal processing and carbonization.

## 2. Charcoal Production System

**Figure 1:**
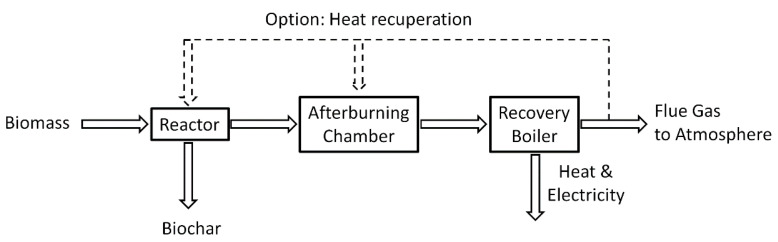
Diagram of the production of charcoal, heat and electricity.

**Figure 2:**
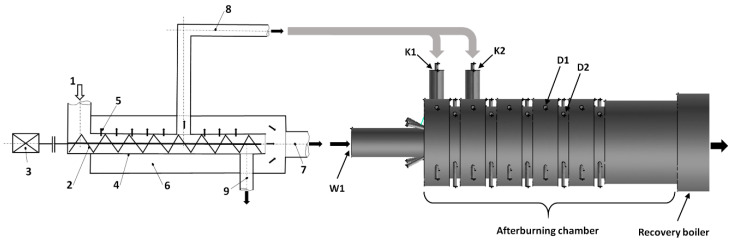
Diagram of the connection between the biomass carbonization reactor with the afterburning chamber and the boiler.

**Figure 3:**
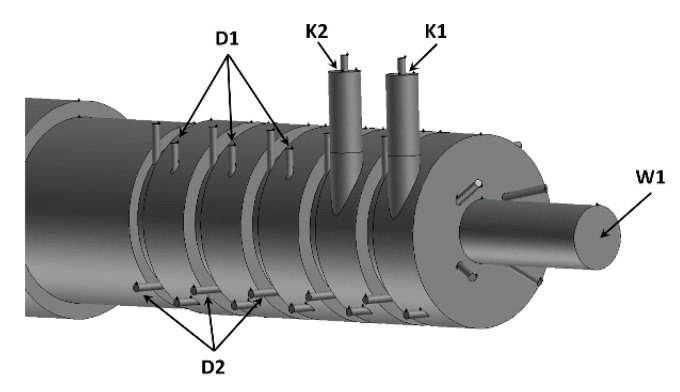
Schematic view of the afterburning chamber.

The system for the production of charcoal, heat and electricity (Figure 1) is composed of a reactor, afterburning chamber and a steam boiler. A diagram of the gasification reactor connected with the afterburning chamber and part of the boiler is shown in Figure 2. Biomass in the form of pellets is supplied into the reactor (Figure 2) through a feeding hopper (1), then, by means of an auger (2) driven by a motor with a gearbox (3), it is transported inside the retort (4), with holes in the upper part (5) allowing for the outflow of gases generated during the heating and devolatilization of biomass to the space (6) where, through a controlled air supply, part of these gases is burned. Due to the design of the reactor and the presence of holes (5) the liquid compounds are evaporated and immediately combusted outside of the retort (4) thus avoiding any condensation of liquids or tar that may bring about serious operational problems. The resulting gases are discharged from the reactor through the main channel (7) together with the flue gases produced during partial combustion into the afterburning chamber through the inlet W1. The rest of the gases are discharged from the reactor through an auxiliary channel (10) allowing direct removal of the generated gases, which are transported to the inlets K1 and K2 in the afterburning chamber. The discharge of gases from the retort (4) through the auxiliary channel (10) allows for limiting the transport of the finest biochar grains with the stream of gases flowing out from the retort through the main channel (7). Furthermore, the quality of the produced biochar discharged through the channel (9) is improved due to the fact that a smaller stream of gases flows over the biochar layer, which may cool down or be absorbed by the biochar. The gases produced in the process are transported through channels (7) and (10) into the ceramic combustion chamber through inlets W1, K1 and K2, which is directly connected to the steam boiler (Figure 1 and Figure 2). The afterburning chamber (Figure 3) has two types of reactor gas inlets: one centrally located in the front wall W1, whose axis coincides with the axis of the afterburning chamber, the other consisting of two channels tangential to the generatrix of the afterburning chamber K1 and K2. The K1 and K2 channels are equipped with nozzles acting as a gas ejector. The afterburning chamber consists of five modules with a variable diameter of 1200 and 1000 mm. The paper analyses the operation of a system consisting of a reactor, a centrifugal afterburning chamber and a steam boiler with a thermal output of 6.5 MW_t_. A pellet flux of 0.956 kg/s with a calorific value of 16.4 MJ/kg is introduced into the reactor in order to obtain the assumed heat flux in steam from the boiler. Physicochemical parameters of the biomass pellet and the biochar are presented in Table 1 (the composition of the biomass and the biochar were determined using a Leco Truspec CHN/S elemental analyzer and the calorimeter IKA C2000 Basic). Pellets moving inside the reactor (Figure 2) are devolatilized as a result of their heating with the gases burned inside the reactor. By properly controlling the rotation of the auger shaft, pellet flowrate and the amount of air fed into the reactor, it is possible to produce charcoal with desired physical and chemical parameters. For the analyzed case 0.261 kg/s charcoal with the parameters listed in Table 1 was discharged from the reactor.

During the process of biomass membrane heating and devolatilization or devolatilization and carbonization in the reactor the gases are produced in the retort. The average composition of the gases is shown in Table 2 (the process temperature was roughly 550–600 °C).

The composition of gases leaving the main channel (7) (cf. Figure 2) changes compared to the composition presented in Table 2. The reason for this is partial combustion of the gases released from the retort (4) through the holes (5) (cf. Figure 2). The composition of the gases leaving the reactor through channel (7) is shown in Table 3. In the latter part of the paper the process of the combustion of the gases generated during biomass carbonization is analyzed and discussed for different conditions and operation of the afterburning chamber.

## 3. Swirl Chamber for Combustion of Pyrolytic Gases

Combustion of gases with low calorific value and varied composition conducted during the production of charcoal is a difficult process. It is necessary to ensure good mixing of flammable gas with the oxidant and to maintain a high temperature of the combustion process in order to increase the rate of chemical reactions. The first condition can be met by using a large number of nozzles to supply oxidizer (air), which allows for obtaining high tangential stresses between the flammable gas and the oxidizer, which in turn accelerates the process of mixing the components. The high temperature of the combustion process can be achieved by building an adiabatic combustion chamber with the smallest possible dimensions.

**Figure 4:**
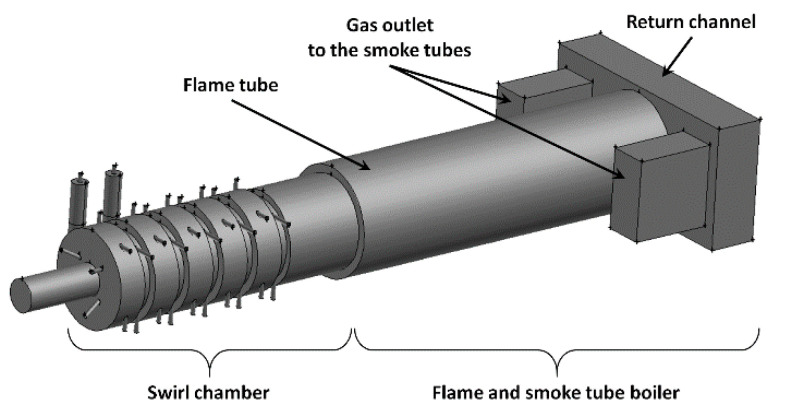
The geometry of the swirl chamber for the afterburning of the pyrolytic gases and some parts of the flame and fire tube boiler.

Based on those assumptions, a combustion chamber was developed for combustion of gases generated in the process of biomass carbonization. The maximum thermal power of the chamber is 6.5 MW_t_. The afterburning chamber consists of five steps with a variable diameter (Figure 3). In the first step, the main inlet of gases from the carbonization reactor W1 is located in the front wall (Figure 2 and Figure 3). Two inlets of pyrolytic gases K1 and K2 are installed in the first and second steps of the afterburning chamber (Figure 3 and Figure 4). The inlets are taken from the auxiliary channel (8) from the carbonization reactor (Figure 2). Each step has a variable diameter, which allows for the accumulation on the created steps (thresholds), of fine charcoal or fly ash lifted together with the gases leaving the reactor. Outside the afterburning chamber, a set of nozzles is installed tangentially to supply air to the combustion process. There are 8 nozzles with a diameter of 50 mm at each step of the afterburning chamber. Four nozzles were placed in each step of the afterburning chamber on a larger diameter (D1) and four nozzles on a smaller diameter (D2) (Figure 3). The swirl combustion chamber is connected to the flame and fire tube boiler (Figure 2 and Figure 4). Figure 4 shows only a part of the boiler: the flame tube, the return channel and the outlet for the fire tubes.

## 4. Numerical Analysis of the Gas Afterburning Chamber Operation

Based on the energy and mass balance of the carbonization reactor, the energy streams, masses and composition of individual streams leaving the carbonization reactor were determined for different conditions of its operation (5 tests). Regardless of the operating conditions, the energy stream contained in the gases flowing into the afterburning chamber through W1, K1 and K2 is identical and ensures a boiler thermal output of 6.5 MW_t_. Based on the composition and flow of flammable gases, the total air flow required for the combustion of gases in the afterburning chamber was determined. Having a stream of flammable gases generated in the reactor, it is possible to supply it all into the afterburning chamber through the main channel W1 (Figure 2 and Figure 3) at a high rate. It is possible to limit this rate by partial feeding the gases to the afterburning chamber through the inlet of K1 and K2 using the installed streams (Figure 2 and Figure 3). With the sets of air supply nozzles (D1, D2) (Figure 2 and Figure 3) installed tangentially on the afterburning chamber, different air supply variants are available for optimal combustion of gases. Based on the above, 5 different cases of calculation of C1 - C5 were prepared, whose detailed parameters are presented in Table 3. It was assumed that with the flow, a 0.5-mm diameter wood charcoal dust stream of 10 g/s is lifted to the afterburning chamber. The temperature of gases leaving the carbonization reactor and entering the afterburning chamber by means of W1, K1 and K2 channels was 600 °C and the gas mass flowrate was roughly 1.01 kg/s. Through the channels K1 and K2, gas with the composition presented in Table 2 flows into the afterburning chamber.

The geometry and the grid used for the computation of the afterburning chamber was developed using the Gambit software (Figure 4) and ANSYS FLUENT 14 software was used for calculations. The developed numerical model of the process allows calculation of the combustion of both pyrolytic gases and the biochar. The calculations were performed using the Reynolds Stress turbulence model, which can also be successfully used for strong swirl flow [8,9,10]. Modelling of flow of biochar dust was based on the discrete phase model, whereas biochar dust and flammable gas combustion was performed using the species transport model, which allows for modelling chemical reactions both in the solid phase and gaseous phase [11,12,13,14,15]. Calculations were based on the radiation model termed discrete ordinate (DO). Calculations of the combustion of biochar dust with average diameter of 0.5 mm were carried out for biochar with physicochemical parameters as presented in Table 1. Reaction rate constants were derived from the studies [11,12].

The process of combustion of gases and biochar dust is described by 6 equations:

- Reaction of oxidation of carbon oxide
  CO + 0.5 O_2_ = CO_2_, (1)
- Reaction of oxidation of fixed carbon (FC)
  C_(s)_ + 0.5 O_2_=CO, (2)
- Boudouard’s reaction
  C_(s)_ + CO_2_ = 2 CO, (3)
- Synthesis of water gas
  C_(s)_ + H_2_O = CO + H_2_, (4)
- Hydrogen oxidation reaction
  H_2_ + 0.5 O_2_ = H_2_O, (5)
- Methane oxidation reaction
  CH_4_ + 1.5 O_2_ = CO + 2H_2_O, (6)

The main purpose of the calculations is to determine the optimum aerodynamic conditions of the combustion process for which flammable components (CO, H_2_, CH_4_) will not be present in the gases leaving the flame tube (flow to the fire tubes). This will make it possible to effectively use the chemical energy contained in the flammable gases leaving the carbonization reactor and limit the emissions of harmful substances into the atmosphere.

## 5. Analysis of the Results of Numerical Calculations

The results of numerical calculations of gas flow and combustion process for 5 analyzed cases C1–C5 are presented in Figure 5, Figure 6, Figure 7, Figure 8, Figure 9, Figure 10, Figure 11, Figure 12, Figure 13, Figure 14, Figure 15, Figure 16, Figure 17, Figure 18, Figure 19, Figure 20 and Figure 21. Figure 5 and Figure 6 show the distribution of the tangential and longitudinal velocity components, respectively.

**Figure 5:**
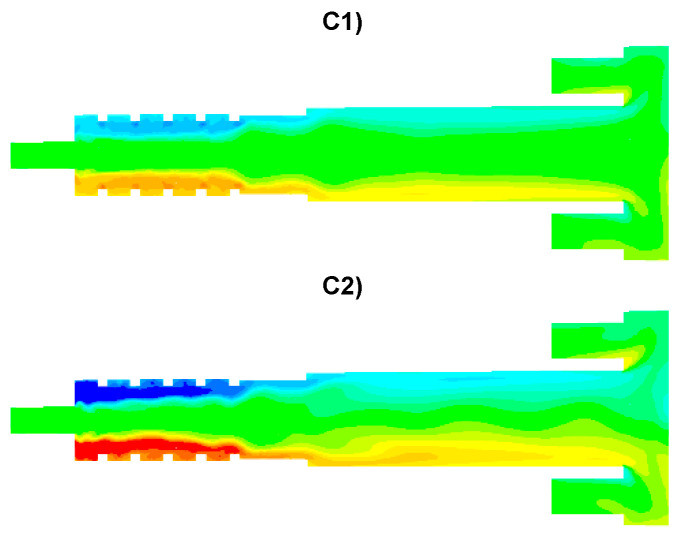

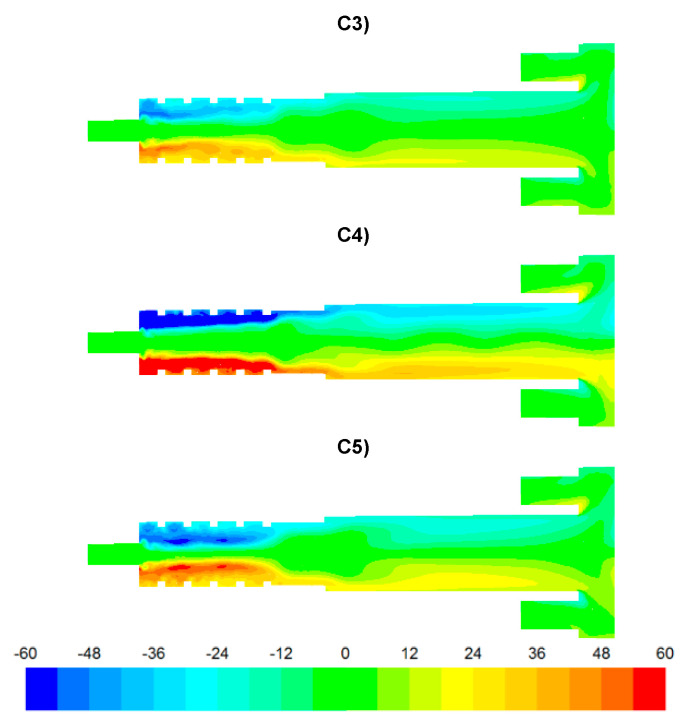
Distribution of tangential velocity component [m/s].

**Figure 6:**
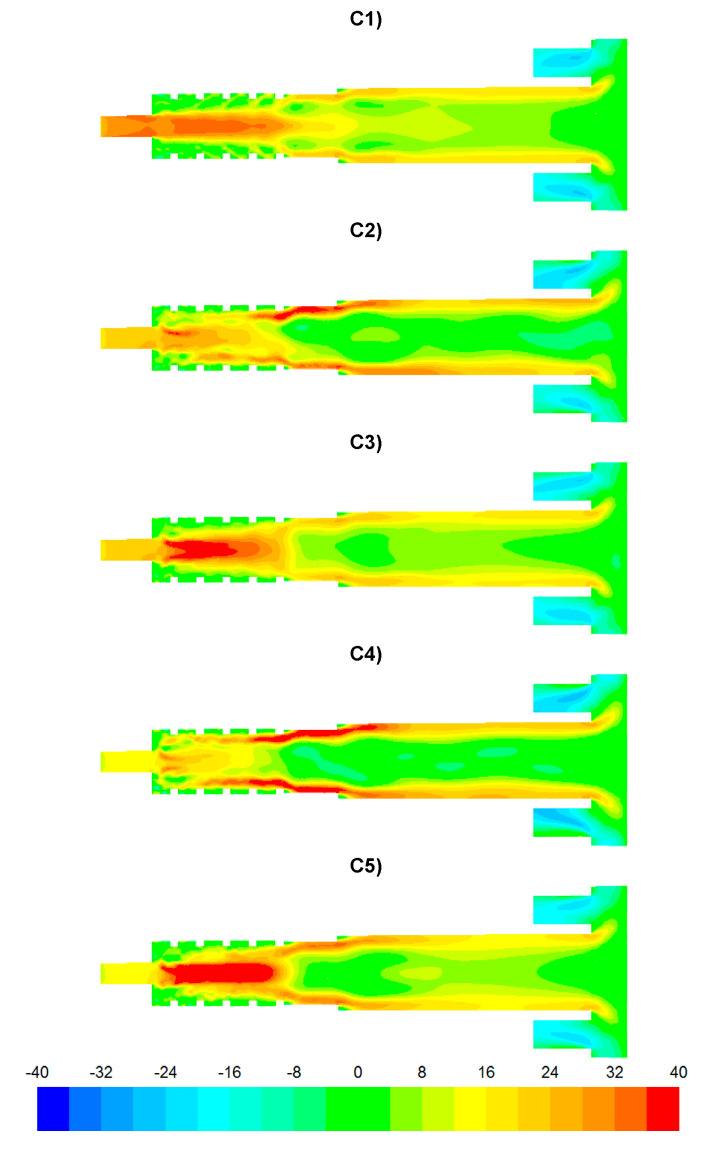
Distribution of longitudinal velocity component [m/s].

Analysis of the distributions of the tangential velocity component (Figure 5) reveals the effect of the tangential air supply to the afterburning chamber clearly visible via the nozzles D1 and D2 (Figure 5). The highest values of the tangential velocity component are located in the afterburning chamber near the walls. The area of strong turbulence is also visible in the vicinity of the boiler flame walls. The intensity of this process depends on the gas velocity (selected variant: Table 3) flowing from the D1 and D2 nozzles. The highest values of the tangential component are found in the cases C2 and C4 when the air is fed through the D1 nozzles at a velocity of 60 m/s. The distributions of the longitudinal velocity component are shown in Figure 6. In the case C1, the highest velocity of the gas flowing from the main channel W1 can be noticed in the central part of the afterburning chamber. For all analyzed cases, both increased longitudinal and tangential velocities are observed in the vicinity of the boiler flame tube walls, which is very beneficial due to the intensification of the heat exchange process from flue gas to water through the flame tube walls.

**Figure 7:**
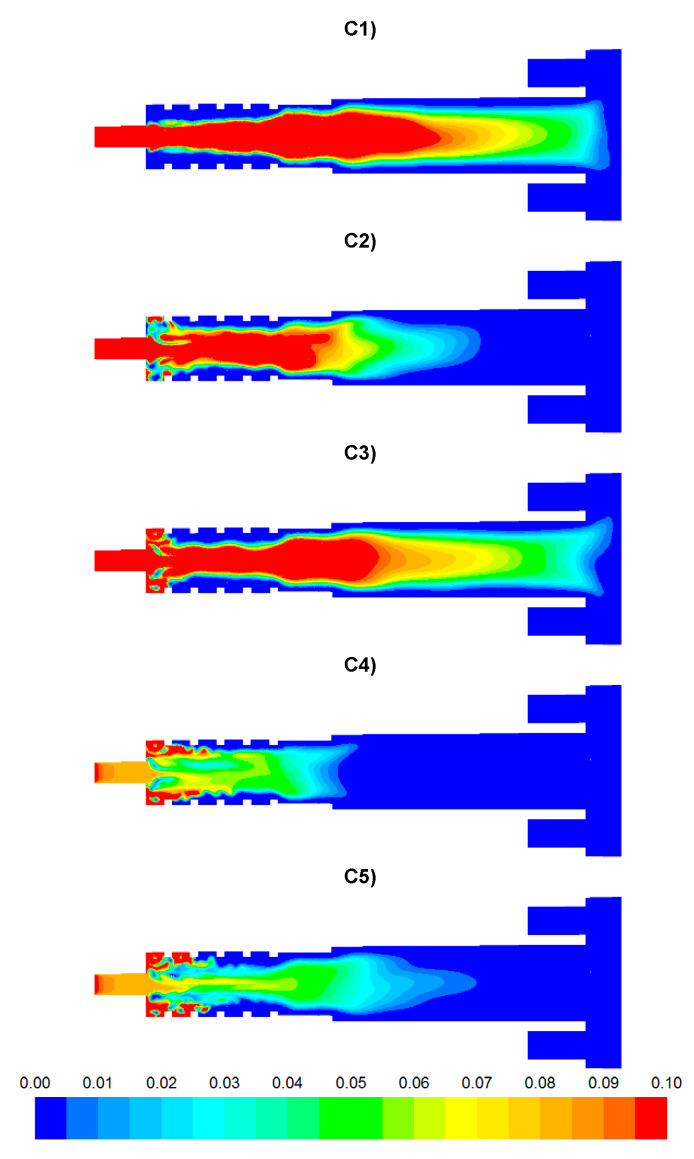
Distributions of CO concentration [-].

**Figure 8:**
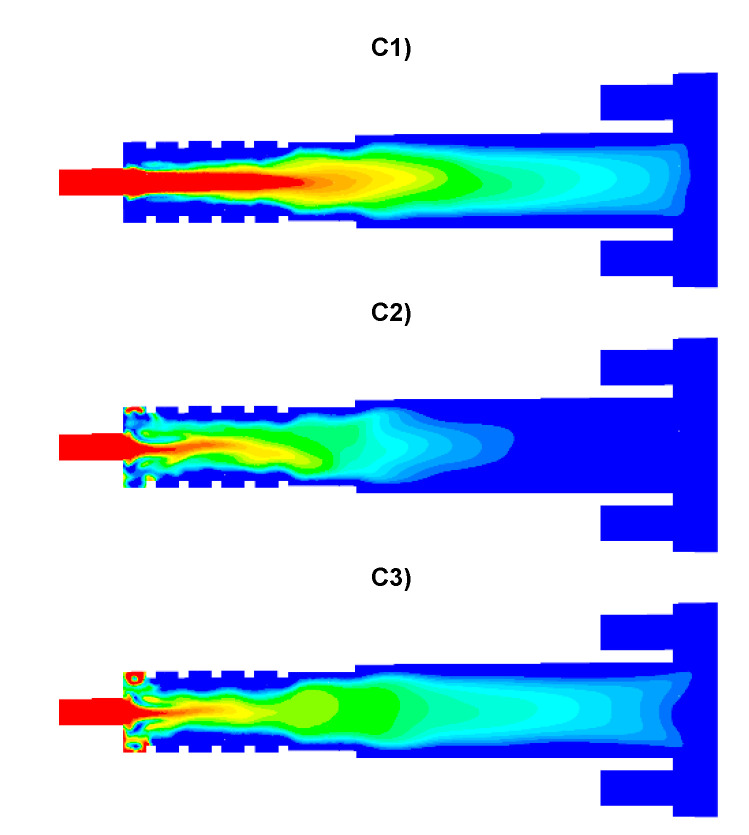

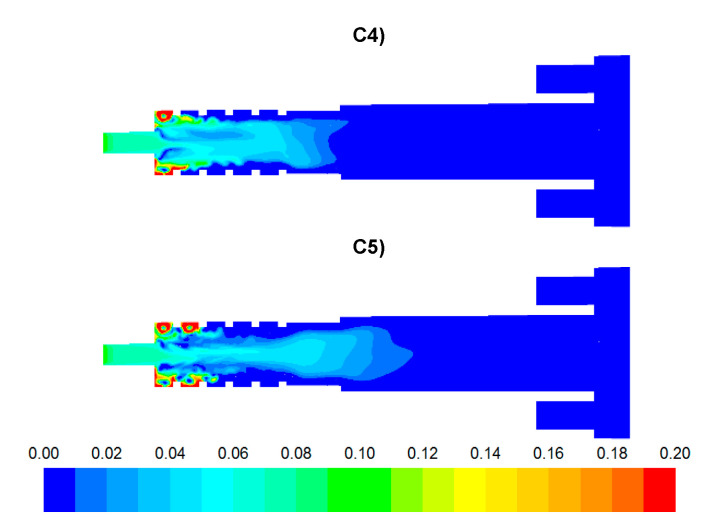
Distributions of H_2_ concentration [-].

**Figure 9:**
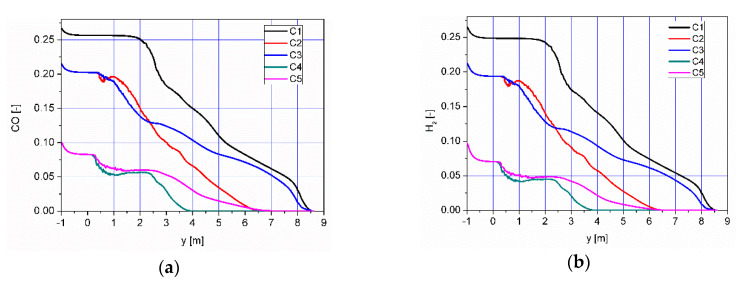
Distributions of concentrations of (**a**) CO and (**b**) H_2_ along the axis of the afterburning chamber and boiler.

**Figure 10:**
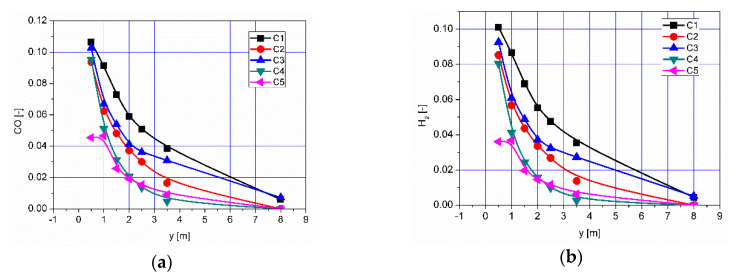
Distributions of the average concentration values of (**a**) CO and (**b**) H_2_ in the selected control planes as shown in Figure 11.

**Figure 11:**
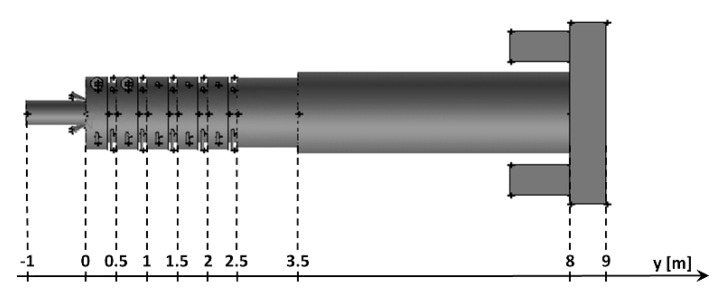
Location of control planes.

Distributions of the concentration of two main combustible components (CO, H_2_) inside the afterburning chamber and the boiler flame tube are shown in Figure 7 and Figure 8. For the assumed scale, the range of both analyzed parameters is very similar. This results directly from the concentration of the introduced components using W1, K1, K2 and the mixing and combustion processes taking place. The area of the highest concentrations (CO and H_2_) covering the virtually entire volume of the afterburning chamber and boiler flame tube is observed for C1 and C3. This is mainly due to the lack of proper mixing and combustion process for these components. For these cases, the above-mentioned flammable components may enter from the boiler flame tube to the return channel at the outlet. The flow of these components to the boiler flame tubes will not allow for afterburning due to the low temperature. This indicates that the mixing and combustion processes for such an organized flow are not optimal. This information is confirmed by the data presented in Figure 9a,b showing the values of CO and H_2_ concentrations along the axis of the afterburning chamber and the boiler. Their high level is observed both in the afterburning chamber and in the boiler flame tube. Furthermore, Figure 10 presents the average values of the concentration of the above-mentioned components in the selected planes located as shown in Figure 11. Analysis of these results reveals that the fastest decrease in the concentration of CO and H_2_ due to the intensive mixing and combustion process occurs in cases C4 and C5, which leads to the conclusion that by using the lowest rate of gas flowing from W1, and thus high rates (fluxes) of flammable gases in K1 and K2, good mixing and quick reaction and combustion of CO and H_2_ can be ensured. For these cases, the concentration of flammable components in the return channel is close to zero (Figure 10). Furthermore, in the cases C1 and C3, CO and H_2_ values ranged from 0.5% to 0.8% (Figure 10).

**Figure 12:**
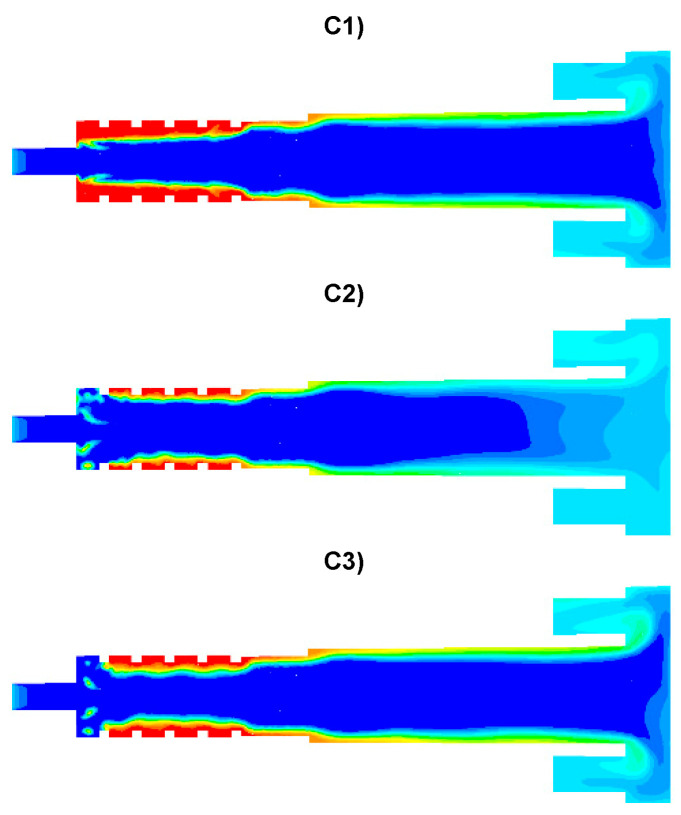

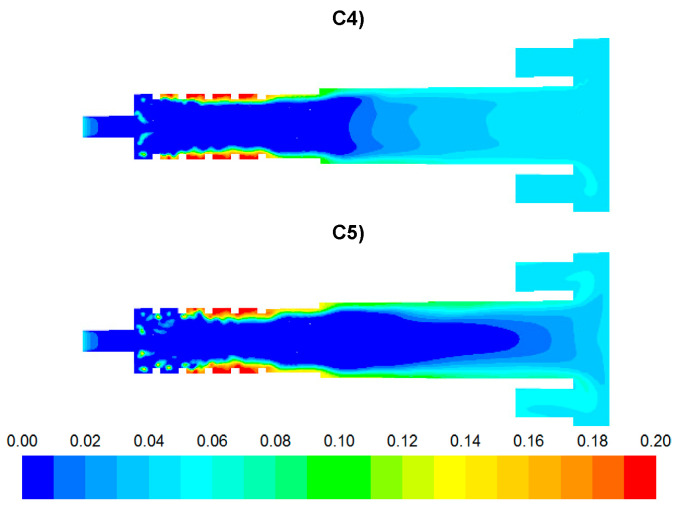
Distributions of O_2_ concentration [-].

Analysis of the distributions of O_2_ concentration indicates that for all cases, the oxygen concentration in the axis of the afterburning chamber is close to zero (Figure 12 and Figure 14a). This results mainly from the composition (Table 3) of the gas that flows into the afterburning chamber through channel W1 and from the tangential air supply (nozzles D1 and D2). With intensive mixing and combustion processes, an increase in oxygen concentration in the axis is observed only in the central part of the flame tube (C4) and in the vicinity of the return channel (C2, C5). Elevated O_2_ concentrations occur near the walls of the afterburning chamber and the boiler flame tube (Figure 12). Regardless of the case of the supply of flammable gases and air to the afterburning chamber, O_2_ concentration in the final section of the flame tube is similar for all the analyzed cases and amounts to ca. 5% (Figure 15a). For the case C4, the concentration of O_2_ in the boiler axis increases (Figure 12), which indicates that combustion processes were completed and the gas is mixed intensively (transport of O_2_ from the vicinity of the boiler walls in the axial direction). This observation is confirmed by the CO and H_2_ distributions for the case C4 (Figure 7, Figure 8, Figure 9 and Figure 10).

**Figure 13:**
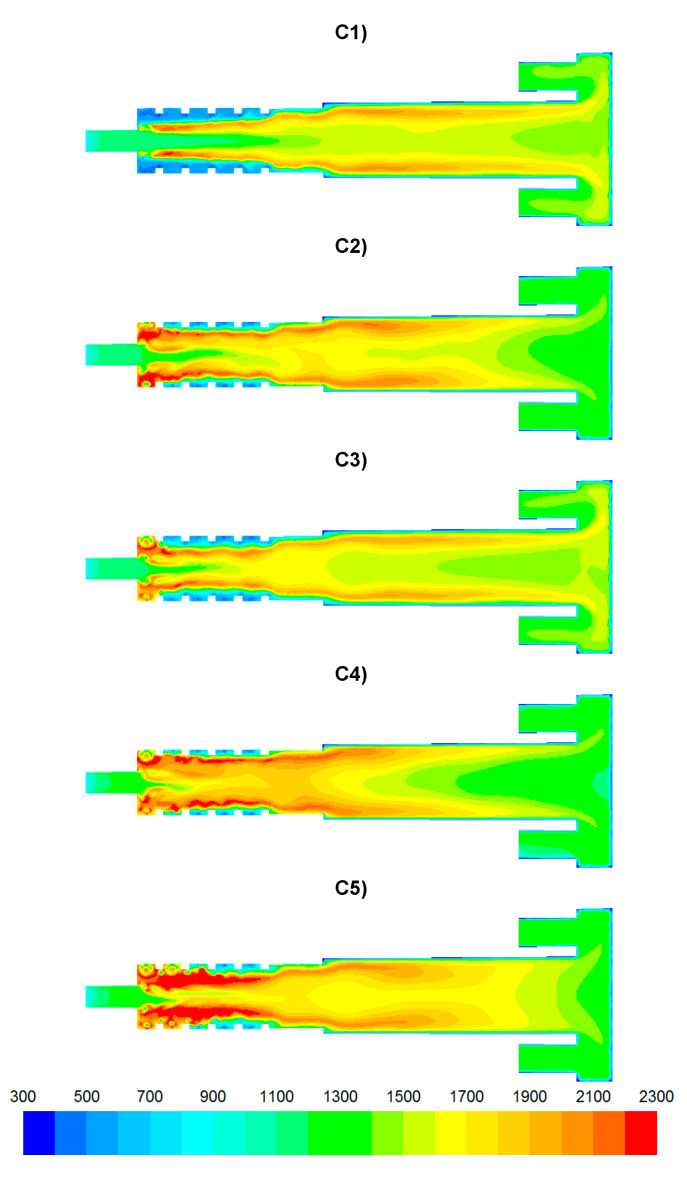
Temperature distributions [K].

**Figure 14:**
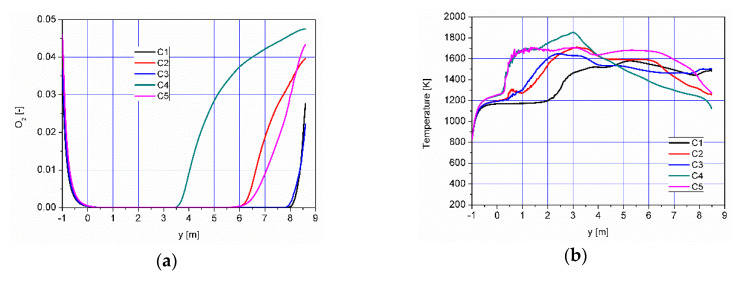
Distributions of O_2_ concentration (**a**) and temperature (**b**) along the axis of the afterburning chamber and the boiler.

**Figure 15:**
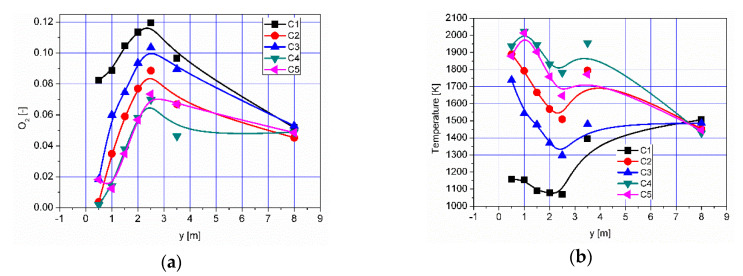
Distributions of the average values of O2 concentration (**a**) and temperature (**b**) in some selected control planes shown in Figure 11.

The temperature distributions for the analyzed cases are presented in Figure 13. The lowest temperatures occurred in the vicinity of the afterburning chamber walls. This is due to the supply of combustion air at a temperature of 300 K by means of nozzles D1 and D2. With intensive mixing processes, the mixture of flammable gases (CO, H_2_) ignites (Figure 7 and Figure 8) and the temperature increases rapidly (Figure 13). In the case C1, temperature values are the lowest among the analyzed cases (Figure 13, Figure 14b and Figure 15b). However, for the cases C4 and C5, due to strong eddy motion and good mixing of flammable gases and oxidant, a noticeable increase in temperature is observed in the initial part of the afterburning chamber. This is due to the way the flammable gases are supplied into the afterburning chamber. For the cases C4 and C5, the largest flammable gas flux is supplied through inlets K1 and K2 (Table 3). Regardless of the variant of the afterburning chamber operation in the final part of the flame tube, the temperature for all analyzed cases reached the level of ca. 1500 K (Figure 15b). The method of supplying flammable gases and oxidant allows for controlling the temperature in the vicinity of the afterburning chamber walls. For the case C1 the low-temperature area in the vicinity of the afterburning chamber walls is clearly visible, resulting from the tangentially supplied oxidant through nozzles D1 and D2 (Figure 12). The lack of flammable gases in the vicinity of the chamber walls (Figure 7 and Figure 8) and high excess oxygen (Figure 12) does not lead to a temperature increase. The change of the method of supplying flammable gases through inlets K1 and K2 causes the intensive mixing of flammable gases with air and thus a shift of the combustion front towards the beginning of the afterburning chamber and a clear increase of temperature in the vicinity of the afterburning chamber walls, which is noticeable for the cases C4 and C5. This character of the combustion process is very beneficial because of the heating of the afterburning chamber walls, whose high temperature stabilizes and accelerates the course of the combustion reaction of individual gaseous components.

**Figure 16:**
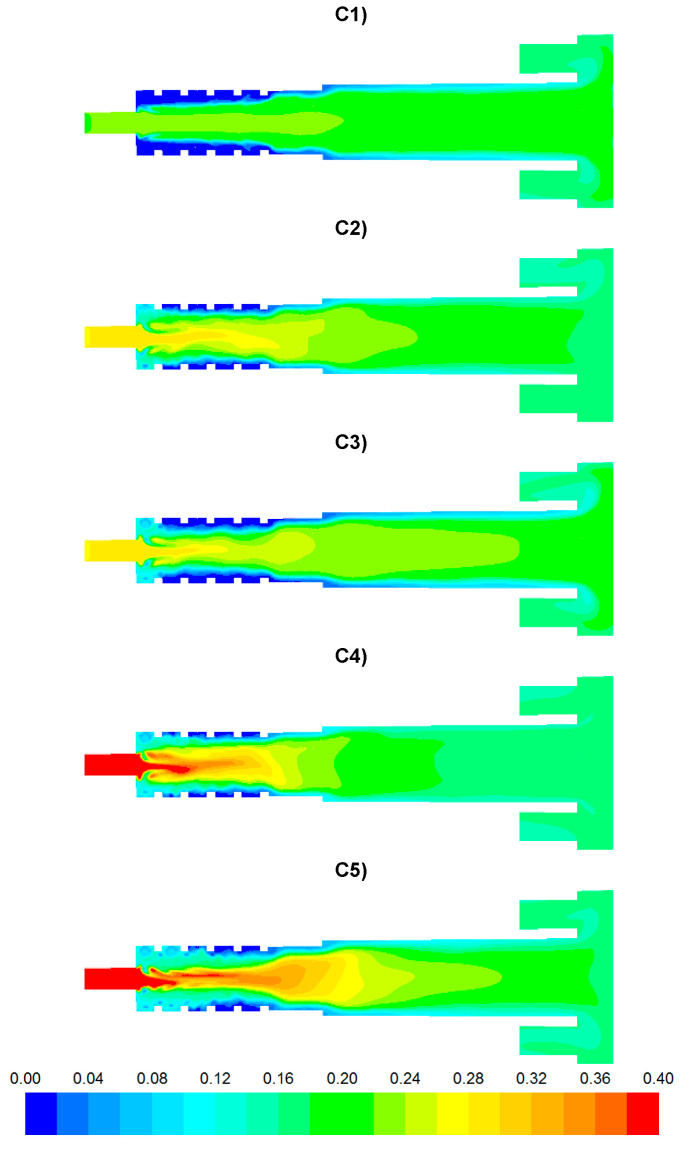
Distributions of CO_2_ concentration [-].

**Figure 17:**
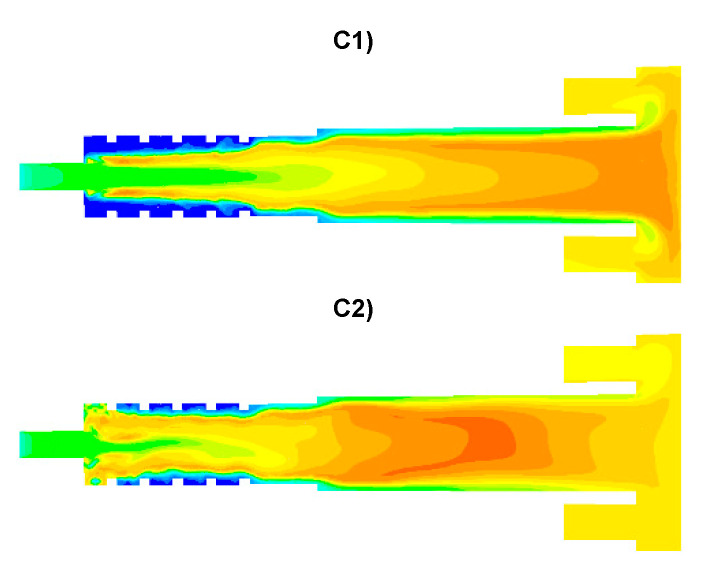

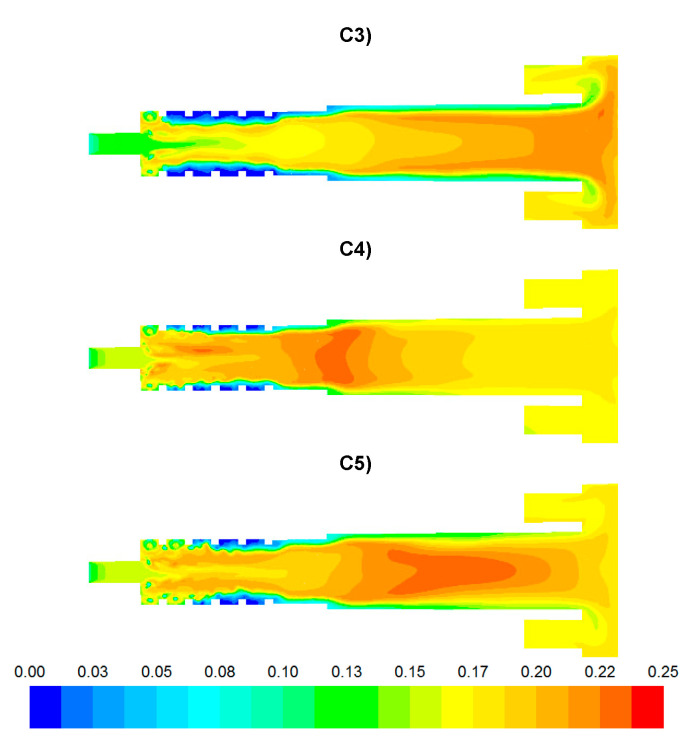
Distributions of H_2_O concentration [-].

**Figure 18:**
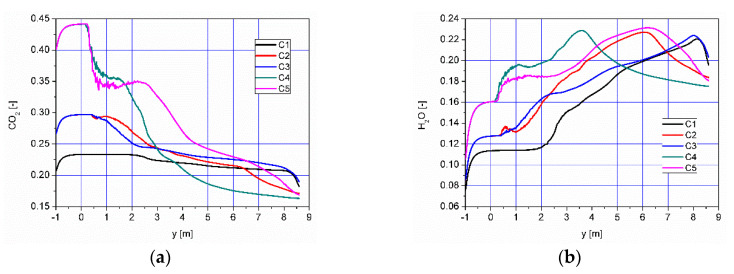
Distributions of concentrations of (**a**) CO_2_ and (**b**) H_2_O along the axis of the afterburning chamber and boiler.

**Figure 19:**
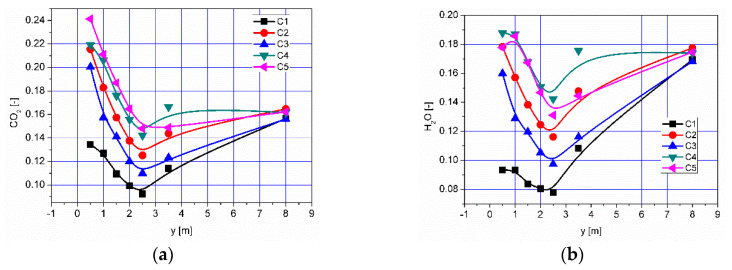
Distributions of the average concentration values of (**a**) CO_2_ and (**b**) H_2_O in the selected control planes as shown in Figure 11.

The effect of the combustion of flammable gases supplied with air into the afterburning chamber (Figure 7 and Figure 8) is the distribution of CO_2_ and H_2_O—the results are presented in Figure 16 and Figure 17, respectively. The highest values of CO_2_ concentration in the afterburning chamber occur in cases C4 and C5 (Figure 16, Figure 17, Figure 18 and Figure 19) and coincide with the area of the highest temperatures (Figure 13). This confirms the conclusion that the combustion front has moved towards the beginning of the afterburning chamber. In the area of the flame tube, the CO_2_ concentration is reduced and balanced through intensive mixing (Figure 18a and Figure 19a). Regardless of the operating conditions of the afterburning chamber, the CO_2_ concentration at the end of the flame tube in the flue gas is about 16% (Figure 19a). Analysis of distributions of H_2_O concentration revealed that the highest H_2_O concentration values were located in the boiler flame tube (Figure 17, Figure 18b and Figure 19b). Regardless of the accepted operating conditions of the afterburning chamber, the H_2_O concentration at the outlet from the flame tube is about 17%.

**Figure 20:**
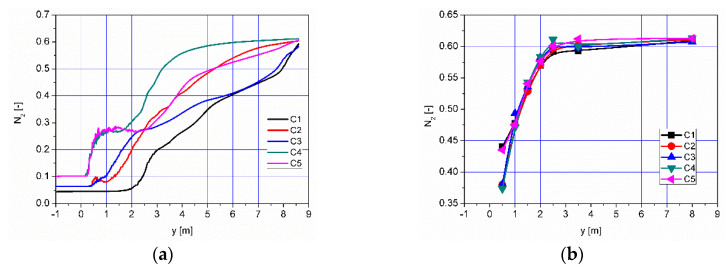
Distribution of (**a**) concentration of N_2_ along the axis of the afterburning chamber and boiler and (**b**) distribution of average values of N_2_ concentration in selected control planes according to Figure 11.

**Figure 21:**
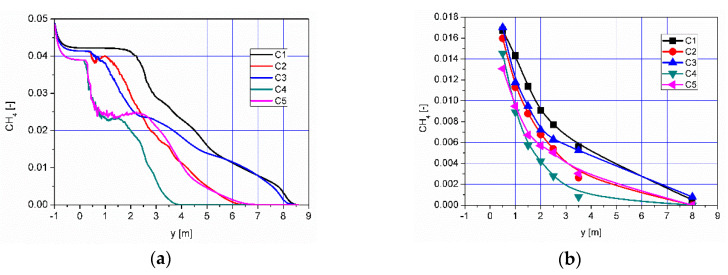
Distribution of (**a**) concentration of CH4 along the axis of afterburning chamber and boiler and (**b**) distribution of mean values of CH4 concentration in selected control planes according to Figure 11.

The distribution of N_2_ concentration in the axis of the afterburning chamber and flame tube is shown in Figure 20a. Continuous increase in N_2_ concentration can be observed, resulting from the gradual air supply in the afterburning chamber. This is confirmed by the distribution of average N_2_ concentration values along the afterburning chamber and the flame tube (Figure 20b). The increase in N_2_ concentration occurs only in the afterburning chamber whereas it remains at a constant level in the flame tube (Figure 21b). For all analyzed cases, the N_2_ concentration at the outlet from the flame tube is about 61%.

The distributions of CH_4_ concentration along the axis of the afterburning chamber and average concentration values in selected planes are presented in Figure 21. It can be observed, similar to the distributions of CO and H_2_ (Figure 7, Figure 8, Figure 9 and Figure 10), that for all analyzed cases, the concentration of CH_4_ decreases with the flow in the afterburning chamber and the boiler flame tube (Figure 21). The decrease in the CH_4_ concentration results from the mixing and combustion processes. Similar to CO and H_2_ analysis, the intensity of these processes is the highest for variants C2, C4 and C5, while the lowest for variants C1 and C3 (Figure 21). 

**Figure 22:**
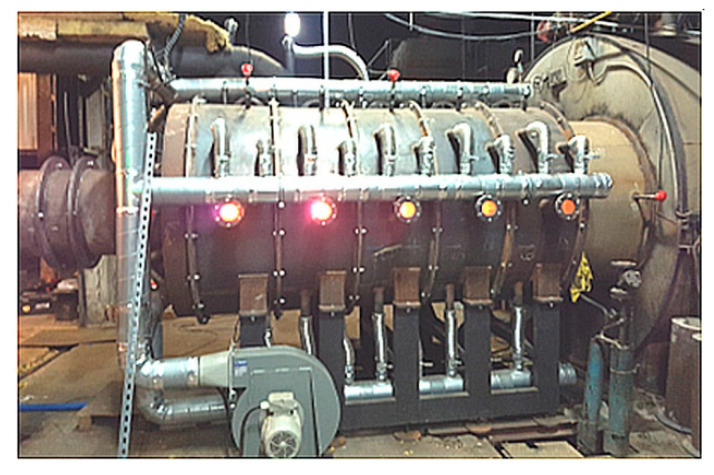
View of the afterburning chamber during operation for the case C5.

The results presented above indicated that the method of ‘supplying’ flammable gases from the biomass carbonization process to the afterburning chamber is critical for good mixing and combustion of individual gas components. Among the five analyzed variants, the most favorable mixing and combustion conditions occurred for the cases C4 and C5. Based on the concept of the biochar production process presented in the study (Figure 1, Figure 2, Figure 3 and Figure 4), an industrial biochar production installation equipped with an afterburning chamber and a steam boiler was constructed and the afterburning chamber is presented in Figure 22. During the operation of the afterburning chamber in the charcoal production installation, preliminary observations confirmed that the most favorable conditions of its operation occurred in the cases C4 and C5. Detailed analysis of the operation of the afterburning chamber and the comparison of numerical calculations results and the experimental data will be the subject of further publications.

## 6. Conclusions

The concept of biomass thermal treatment that has been presented in the current paper allows for the production of heat and valuable solid product (biochar) that may be then used, e.g., as a fertilizer ingredient, soil improver, filter material, cosmetic improver, or as a high-quality energy carrier. During the thermal treatment the resulting flammable gases are partly used to maintain the process of biomass carbonization but the vast majority of them must be burned outside the carbonization reactor in a dedicated afterburning chamber.

Since the production of the biochar requires management of the flammable gases it is necessary to design the afterburning chamber in such a manner that it provides optimal conditions for the completion of this process. Due to the possibility of changing the inflow of flammable gases to the combustion chamber (inlets K1 and K2) and strong turbulence of the flow (nozzles D1 and D2) it is possible to create process conditions that will ensure complete afterburning of individual flammable components. The calculations have shown that the cases C4 and C5 allow for ensuring optimal combustion conditions in the carbonization reactor. Preliminary observations of the combustion process in the afterburning chamber (Figure 22) were promising and confirmed the correctness of the calculations and good results for the cases C4 and C5. As a next step, we plan to carry out detailed calculations of the combustion process for the cases C4 and C5 and compare the results with measurements of selected physical quantities at the real facility (Figure 22). The data will allow for accurate calibration of the numerical model of the combustion process in the afterburning chamber.

## Figures and Tables

**Table 1:** Parameters of biomass (sunflower husk pellets) and biochar (the values are given in dry state).

	Pellet	Biochar
C [%]	49.32	85.61
H [%]	6.11	2.61
N [%]	0.36	0.85
S [%]	0.07	0.05
O [%]	41.54	1.55
A [%]	2.60	9.33
W [%]	9.40	0
LHV [MJ/kg]	16.4	31.9

**Table 2:** Average composition of gases produced during biomass devolatilization at 550–600 °C.

Component	[-]
O_2_ [-]	0.025
CO_2_ [-]	0.05
H_2_O [-]	0.05
CO [-]	0.4
H_2_ [-]	0.4
CH_4_ [-]	0.05
N_2_ [-]	0.025

**Table 3:** List of basic process parameters for individual calculation cases (the air ratio for all cases was 1.3).

	C1	C2	C3	C4	C5
**W1** [m/s]	22.5	16.25	16.25	10	10
**K1** [m/s]	0	25	25	50	25
**K2** [m/s]	0	0	0	0	25
**D1** [m/s]	0	60	30	60	30
**D2** [m/s]	60	0	30	0	30
The composition of gas flowing from the reactor through the main channel **W1**					
O_2_ [-]	0.038	0.043	0.043	0.056	0.056
CO_2_ [-]	0.219	0.285	0.285	0.444	0.444
H_2_O [-]	0.076	0.087	0.087	0.111	0.111
CO [-]	0.283	0.231	0.231	0.111	0.111
H_2_ [-]	0.283	0.231	0.231	0.111	0.111
CH_4_ [-]	0.053	0.054	0.054	0.056	0.056
N_2_ [-]	0.048	0.069	0.069	0.111	0.111
